# Thrombolyse d’un AVC ischémique vertébro-basilaire à N’Djamena, République du Tchad

**DOI:** 10.11604/pamj.2018.29.35.14547

**Published:** 2018-01-16

**Authors:** Antoine Lamblin, Marc Bascou, Eve Drouard, Nicolas Alberti, Thierry de Greslan

**Affiliations:** 1 Service d’Anesthésie-Réanimation, Hôpital d’Instruction des Armées Percy, Clamart, France; 2 Centre Médical des Armées, Lyon, France; 3 Service de Neurologie, Hôpital d’Instruction des Armées Percy, Clamart, France; 4 Service de Radiologie, Centre Hospitalier Alpes-Léman, Contamine-sur-Arve, France

**Keywords:** Ischemic stroke, thrombolysis, Africa, Accident vasculaire cérébral ischémique, thrombolyse, Afrique

## Abstract

L’accident vasculaire cérébral ischémique est une pathologie rare chez les militaires français, mais les médecins militaires projetés en opérations extérieures peuvent être amenés à en prendre en charge, notamment en Afrique Sub-Saharienne. Dès lors, il s’agit d’une urgence vitale nécessitant de réagir rapidement avec des moyens limités, de façon multidisciplinaire avec les médecins neurologues de France métropolitaine, afin d’assurer au patient une prise en charge optimale. Nous rapportons le cas d’un patient victime d’un accident vasculaire cérébral ischémique sur le territoire vertébro-basilaire, traité par thrombolyse intraveineuse.

## Abstract (en)

Ischemic stroke is rare among the French Armed Forces, but Army doctors on missions may be called on to manage it, in particular in sub-Saharan Africa. Therefore, this is a critical emergency requiring early multidisciplinary approach, with limited means, involving neurologists from mainland France, in order to ensure patient optimal treatment. We here report the case of a patient with vertebro-basilar ischemic stroke treated with intravenous thrombolysis.

## Introduction

La prise en charge des accidents vasculaires cérébraux ischémiques (AVCi) est une urgence absolue et doit être réalisée en priorité dans des unités neuro-vasculaires. Le traitement repose en premier lieu sur la thrombolyse intraveineuse après réalisation d’une imagerie par résonnance magnétique qui objective l’AVCi. En milieu isolé ou dans les pays non pourvus de telles structures, le traitement est souvent symptomatique. La thrombolyse intraveineuse pourra être réalisée uniquement dans des cas exceptionnels après avoir éliminé des contre-indications à ce traitement en évaluant scrupuleusement le rapport bénéfices/risques et seulement après concertation auprès d’un médecin neurologue habitué à gérer les urgences neuro-vasculaires.

## Patient et observation

Nous rapportons le cas d´un patient âgé de 50 ans, militaire français en séjour depuis 2 ans au Tchad, sans antécédents notables, qui consultait en urgence au poste de santé unique (PSU) de la base Adji Kossei de N´Djamena pour l´apparition brutale d´un déficit neurologique. Ses facteurs de risque cardiovasculaires étaient un tabagisme estimé à 10 paquets-années et un léger surpoids (indice de masse corporelle à 28 kg/m^2^). Vers 12h, alors qu’il travaillait devant son écran d’ordinateur, il présentait des troubles visuels associés à des troubles de l’équilibre. Il était pris en charge vers 13h. La tension artérielle était à 142/81 mmHg, symétrique, la SpO2 à 97% en air ambiant, le pouls à 75 battements par minute, la température centrale à 37,6°C. L’examen neurologique retrouvait un score NIHSS (National Institute of Health Stroke Score) à 2 avec une diplopie monoculaire de l’œil gauche (sans troubles oculomoteurs), associée à des troubles de l’équilibre en position debout, sans vertiges. Les réflexes du tronc cérébral étaient conservés et il n’était pas retrouvé de troubles sensitivo-moteur, de nystagmus ou d’atteinte des paires crâniennes. Le reste de l’examen était sans particularité.

Sur le plan biologique, la glycémie était à 5.5 mmol/l, la natrémie à 129 mmol/l. La NFS et le bilan de coagulation étaient sans anomalie. Le test de détection rapide pour le paludisme était négatif (détection de l’Histidine Rich Protein 2 ou HRP 2). L’ECG s’inscrivait en rythme sinusal et régulier, sans troubles de la conduction ou de la repolarisation. La fraction d’éjection du ventricule gauche était normale à l’échographie cardiaque trans-thoracique (ETT), sans atteinte de la fonction cinétique segmentaire ni valvulopathie visibles. Un scanner cérébral sans injection, ainsi qu’un angioscanner des troncs supra-aortiques (TSA) était alors immédiatement réalisé, interprété conjointement par le médecin urgentiste et le médecin anesthésiste-réanimateur du PSU (devant l’absence de médecins radiologue et neurologue sur place) qui ne retrouvait pas d’hémorragie aiguë ni de thrombus évident sur le territoire vertébro-basilaire. Devant la forte suspicion clinique d’AVCi sur le territoire vertébro-basilaire, et en l’absence de moyen de réaliser une imagerie par résonnance magnétique (IRM), il était décidé, après avis téléphonique auprès d’un médecin neurologue de métropole, de procéder à la réalisation d’une thrombolyse intraveineuse par ténectéplase 50 mg (10000 UI) vers 14h20, soit 2h20 après le début de l’apparition des symptômes.

Vers 15h, il était noté une disparition complète des symptômes neurologiques et une récupération *ad integrum*, sans apparition de signes hémorragiques. Le patient était ensuite évacué en urgence par moyen aérien stratégique vers un hôpital militaire de métropole où le bilan étiologique était complété. La ponction lombaire était sans anomalie décelable. Deux IRM de diffusion réalisées respectivement 24 et 48 heures après l’apparition des symptômes ne retrouvaient pas de dissection des troncs supra-aortiques ni de séquelles ischémique sur la fosse postérieure. L’échographie cardiaque trans-oesophagienne ne montrait qu’un athérome minime de la crosse aortique, et l’enregistrement sur 24h de l’électrocardiogramme (Holter-ECG) réalisé à plusieurs reprises ne montrait pas de troubles du rythme ou de la conduction.

Un traitement préventif par acide acétylsalicylique 75 mg/j associé à une statine était initié. Les consultations de suivi permettaient d’exclure toute récidive de symptômes neurologiques. Une inaptitude temporaire aux opérations extérieures et aux séjours outre-mer d’un an était également décidée.

## Discussion

Les AVC vertébro-basilaires représentent 20% des AVCi environ. Les symptômes cliniques sont très variables du simple vertige à un coma profond par ischémie du tronc cérébral [1]. La plupart des patients ont des prodromes à type de céphalées, de vertiges, mais aussi des bâillements ou une somnolence, une altération du champ visuel ou une diplopie, une hémiparésie, des troubles de la sensibilité, des troubles de l´équilibre, une dysphagie, une dysarthrie, une parésie faciale, des « drop attacks » [2]. La survenue des signes d´AVC peut être brutale ou plus fréquemment progressive avec une aggravation sur plusieurs heures. Avec ou sans recanalisation artérielle, l´ischémie et l´oedème cérébelleux sont des complications fréquentes des AVC vertébro-basilaires [3]. Le diagnostic de ce type d’AVC est souvent difficile et repose avant tout sur l’examen clinique et l’interrogatoire, comme c’était le cas dans cette observation. L’imagerie doit être réalisée en urgence, dans un délai de moins de 25 minutes après l’admission du patient, l’objectif étant d’éliminer une hémorragie, de confirmer et préciser l’étendue de l’infarctus cérébral, d’identifier le siège de l’occlusion artérielle ainsi que l’étendue de la pénombre ischémique [4]. L’Imagerie par Résonnance Magnétique (IRM) est la technique privilégiée pour répondre à ces objectifs. En cas d’indisponibilité de l’IRM, un scanner cérébral doit être réalisé, comportant une acquisition sans injection, un angioscanner du polygone de Willis et éventuellement un scanner de perfusion.

Dans le cas que nous présentons, seuls une acquisition sans injection et un angioscanner des troncs supra-aortiques étaient réalisés. En effet, aucun appareil d’IRM n’était disponible sur la ville de N’Djamena au moment de la prise en charge du patient. En l’absence de médecin radiologue disponible sur place, il n’a pas non plus été possible de réaliser de scanner de perfusion, rendant sa réalisation technique et son interprétation impossible par des médecins non spécialisés en radiologie. Un autre problème rencontré dans ce cas est celui de la lecture des images du scanner cérébral conjointement par le médecin anesthésiste-réanimateur et le médecin urgentiste, sans aucun moyen rapide de transmission des images à un médecin radiologue ou un médecin neurologue en France métropolitaine. Le seul contact possible étant téléphonique, c’est donc sur les seules constations des médecins présents sur place, après contact avec un médecin neurologue de métropole que la décision de thrombolyse intraveineuse a été prise, en raison de l’absence de saignement intracrânien visible sur le scanner cérébral. Des moyens de télétransmission des images sécurisés, fiables et rapides vont prochainement être mis en place au PSU de la base Adji Kossei de N’Djamena, permettant à l’avenir de discuter de la prise en charge de ce type de patients en lien avec les médecins radiologues et neurologues des Hôpitaux d’Instruction des Armées (HIA) de métropole.

Chez ce patient, le diagnostic initial d’AVCi vertébro-basilaire a été confirmé à distance en service de neurologie en France uniquement sur les données anamnestiques et cliniques et non sur les résultats des deux IRM réalisées respectivement 24h et 48h après l’apparition des symptômes neurologiques. La faible puissance de l’IRM utilisée (1,5 Tesla), ainsi que l’absence de coupes fines peut expliquer l’absence de signes d’AVCi à distance de la thrombolyse. La réalisation d´un examen avec coupes fines, en séquence de diffusion B2000 aurait peut-être pu permettre la mise en évidence d´infarctus de très petit volume non visualisé par IRM cérébrale conventionnelle [5]. Une autre donnée à considérer est la normalisation potentielle des images de l’IRM à distance de la thrombolyse qui a été réalisée précocement dans ce cas, n’entrainant potentiellement aucune séquelle ischémique cérébrale.

L´objectif du traitement des AVC ischémiques est d’assurer une reperfusion la plus rapide possible du cerveau. Le délai de revascularisation est l´élément le plus important du pronostic et doit être le plus court possible, afin de limiter le temps d’ischémie cérébrale. Plusieurs techniques sont décrites: la thrombolyse intraveineuse, la thrombolyse intra-artérielle et la thrombectomie mécanique (chirurgicale ou endartérielle) [6]. La seule technique envisageable au PSU du camp Kossei de N’Djamena était la thrombolyse intraveineuse qui est le traitement de référence de l´AVC ischémique et dont l’on bénéficie du plus de recul. L’indication de thrombolyse dans le cas présenté ici était discutée avec les neurologues de métropole en évaluant de manière scrupuleuse le bénéfice attendu à savoir d’éviter une diplopie séquellaire qui aurait pu être préjudiciable pour le patient compte-tenu de son âge et de sa profession mis en balance avec le risque hémorragique potentiel d’une lésion non diagnostiquée au scanner cérébral (par exemple un cavernome de la fosse postérieure).

Peu de cas de thrombolyse intraveineuse sont rapportés dans la littérature en Afrique Sub-Saharienne, en particulier en raison de l’éloignement des grandes villes, des délais par rapport au début des symptômes dépassés, de l’absence d’unités spécialisées (unités neuro-vasculaires) et de matériel d’imagerie adapté [7]. C’est néanmoins l’altéplase (rtPA) à la dose de 0,9 mg/kg (dose maximale de 90 mg) qui doit être administrée dans cette indication, dans les 3 heures qui suivent la survenue des premiers signes d’AVC, après s’être évidemment assuré de l’absence de contre-indications. L’utilisation hors autorisation de mise sur le marché du ténéctéplase à la posologie de 0.25 mg/kg (dose maximale de 25 mg) est également possible et a été étudiée en comparaison avec l’utilisation de l’altéplase, avec des résultats similaires en termes d’efficacité et de risque hémorragique [8]. Il existe cependant un risque d’hémorragie cérébrale augmenté chez les patients recevant une posologie plus importante que 0.25 mg/kg, comme utilisée pour le patient que nous avons pris en charge [9]. Ceci est dû à une méconnaissance de ce médicament dans l’indication de thrombolyse intraveineuse d’AVCi et mérite d’être souligné.

## Conclusion

La prise en charge d’un AVCi en opérations extérieures en Afrique Sub-Saharienne est un réel défi pour les médecins urgentistes et réanimateurs militaires et doit faire l’objet d’une discussion multidisciplinaire avec les médecins neurologues et radiologues de France métropolitaine. La réalisation d’une imagerie cérébrale par tomodensitométrie a permis dans ce cas de réaliser une thrombolyse intra-veineuse par ténectéplase, ayant entraîné une régression quasi-immédiate des symptômes neurologiques, après évaluation scrupuleuse du rapport bénéfice/risques. La posologie de cette molécule doit cependant être adaptée à la dose de 0.25 mg/kg. La mise en place de moyens modernes de télétransmission au PSU de la base Adji Kossei de N’Djamena permettra à l’avenir une meilleure prise en charge de ce type de pathologies.

## Conflits d’intérêts

Les auteurs ne déclarent aucun conflit d’intérêts.
